# B-cell subpopulations in children: National reference values

**DOI:** 10.1002/iid3.26

**Published:** 2014-07-31

**Authors:** Marie Duchamp, Delphine Sterlin, Aminata Diabate, Béatrice Uring-Lambert, Valérie Guérin-El Khourouj, Brigitte Le Mauff, Delphine Monnier, Christophe Malcus, Myriam Labalette, Capucine Picard

**Affiliations:** 1 Study Center of Primary Immunodeficiencies, Assistance Publique-Hôpitaux de Paris (APHP), Necker HospitalParis, France; 2 Laboratory of Immunology, Nouvel Hôpital CivilStrasbourg, France; 3 Laboratory of Immunology, APHP—Hôpitaux de Paris, Robert Debré HospitalParis, France; 4 Laboratory of Immunology, Caen Hospital, Université de Caen Basse NormandieCaen, France; 5 Laboratory of Immunology, Cell Therapy and Hematopoiesis, Pontchaillou HospitalRennes, France; 6 Laboratory of Immunology, Hospices Civils de Lyon, Edouard Herriot HospitalLyon, France; 7 Laboratory of Immunology, Lille HospitalLille, France; 8 Laboratory of Human Genetics of Infectious Diseases, Necker Branch, INSERM UMR 1163, Necker Medical School, Imagine Institute, Paris Descartes University—Sorbonne Paris CitéParis, France; 9 Centre de référence des déficits immunitaires héréditaires (CEREDIH), APHP, Necker HospitalParis, France

**Keywords:** B-cells, children, primary immunodeficiency, reference values

## Abstract

Peripheral B-lymphocytes undergo a series of changes during the first few years of life. Encounters with foreign antigens lead to maturation and differentiation. Several primary antibody deficiencies (PADs) affecting B-cell development are associated with abnormalities in the composition and/or differentiation of B-cell compartments. The most recent international classifications of primary immunodeficiencies (PIDs) and common variable immunodeficiencies (CVID) have highlighted the importance of B-cell immunophenotyping and age-specific reference intervals for diagnostic purposes. We established national reference values for memory B-cell subpopulations, on the basis of CD27 and surface IgD expression in the peripheral blood of 242 healthy children. We report here the absolute counts and percentages of naive, switched and non-switched memory B-cells for seven age groups, from neonates to adults. We found that the naive B-cells percentage declined between the ages of 6 months and 8 years, after which it remained stable at about 70–80%. Memory B-cells are already present at birth and their numbers increase throughout childhood, stabilizing between the ages of 12 and 18 years. The definition of reference intervals for pediatric B-cell levels should facilitate the screening and diagnosis of various B-cell immunodeficiencies. This multicenter study, providing national reference values, should thus facilitate immunological diagnosis in children.

## Introduction

B-cells play a central role in humoral immunity, particularly that directed against extracellular infectious pathogens [1,2]. Indeed, antibodies are the only mechanism of adaptive immunity capable of preventing infections from becoming established [3]. The B-lymphocyte lineage undergoes a maturation process resulting in considerable plasticity of the antibody response. The early stages of B-cell development take place in the bone marrow, after which, the B-cells continue to mature in peripheral lymphoid organs, where they encounter foreign antigens [4]. Antigenic stimulation triggers the proliferation and differentiation of antigen-specific cells. Successive steps in B-cell differentiation are characterized by an ordered molecular program and stochastic immunoglobulin (Ig) gene rearrangements and mutations, resulting in the generation of two types of affinity-matured B-cells: memory B-cells and antibody-secreting plasma cells [5,6]. Memory B-cells circulate continuously between the blood and lymphoid organs, and can differentiate rapidly into effector cells upon cognate antigen recognition, whereas the long-lived plasma cells reside in the bone marrow and produce high-affinity antibodies without antigenic stimulation [7–9]. Simultaneous studies of the levels of the human memory B-cell marker, CD27, and of the surface expression of IgD, in the B-cell compartments of blood have identified different stages of maturation during the course of life [10,11]. The most obvious changes in the composition of the peripheral B-cell pool occur in the first 5 years of life [12]. Indeed, all memory B-cell subpopulations increase in size during the first few years of life, following encounters with foreign antigens. At birth, CD27^+^ IgM^+^ IgD^+^ memory B-cells constitute the largest subpopulation of the memory B-cell compartment, but the size of this subpopulation decreases during childhood and then stabilizes in young adults. By contrast, CD27^+^ class-switched B-cells, which have lost their surface expression of IgD, gradually increase in number during aging [12–14].

Changes in antibody production are the hallmark of various human B-cell deficiencies known as primary antibody deficiencies (PADs); these conditions are the most common primary immunodeficiencies (PIDs) [15]. Several B-cell deficiencies result from abnormalities of B-cell development [15,16]. A flow cytometric approach, defining B-cell phenotypes, has been successfully used to classify circulating B-cell abnormalities in patients with PIDs [17]. Indeed, patients with agammaglobulinemia caused by hemizygous *BTK* mutations generally lack CD19 cells [18,19], whereas patients with ICF (immunodeficiency, centromeric instability, facial abnormalities) syndrome caused by an autosomal recessive genetic defect in *DNMT3B* or *ZTB2B4* display a profound selective memory (IgD^−^ CD27^+^) B-cell defect [20]. In common variable immunodeficiency (CVID), a heterogeneous group of PIDs, almost all patients have impaired switched-memory B-cells [21–23]. A European classification has been put forward in which subgroups of CVID patients are defined on the basis of the percentages of transitional and memory B-cells in adults [24]. Since 2008, several studies have highlighted the importance of age-specific reference intervals for the correct interpretation of B-cell subpopulation data from children for diagnostic purposes [25–29]. However, only adult classifications currently exist, and these must be adapted to the maturation state of the immune systems of children of various ages [25–29]. We established national reference values for B-lymphocyte subpopulations in the peripheral blood of healthy children. The findings of this multicenter study should make it possible to analyze large cohorts of individuals, ranging from neonates to adults. These national pediatric reference intervals will be useful for the design of new studies including sufficient patients for the evaluation of diagnostic or classification criteria.

## Materials and Methods

### Study cohort

Between June 2012 and November 2012, 292 healthy children aged 0–18 years (mean age: 6.44 years) were enrolled in this study. Children with suspected or confirmed HIV infection, PID, active infection, or on immunosuppressive treatment or with a chronic disease that might affect the immune system were excluded. These healthy children were referred to the outpatient clinics of seven French hospitals (Strasbourg Hospital, Rennes Hospital, Lyon Hospital, Caen Hospital, Lille Hospital, Necker—Enfants Malades Hospital and Robert Debré Hospital, Paris) for diagnostic blood testing. Most underwent routine blood testing before minor surgical or diagnostic procedures. All the immunological laboratories participating in this study belong to the national network, CEREDIH. Peripheral venous blood samples were collected into ethylenediamine tetraacetic acid (EDTA) to prevent coagulation and processed within 24 h. We determined C-reactive protein concentration and counts of leukocytes and lymphocytes, to confirm the absence of biological abnormalities in the individuals included in this study. Abnormal counts of leucocytes or lymphocytes and/or increased levels of C-reactive protein according to the laboratory reference values were excluded of the statistical analysis. The study was performed in accordance with the modified version of the Helsinki Declaration.

### B-cell immunophenotyping

**Figure 1:**
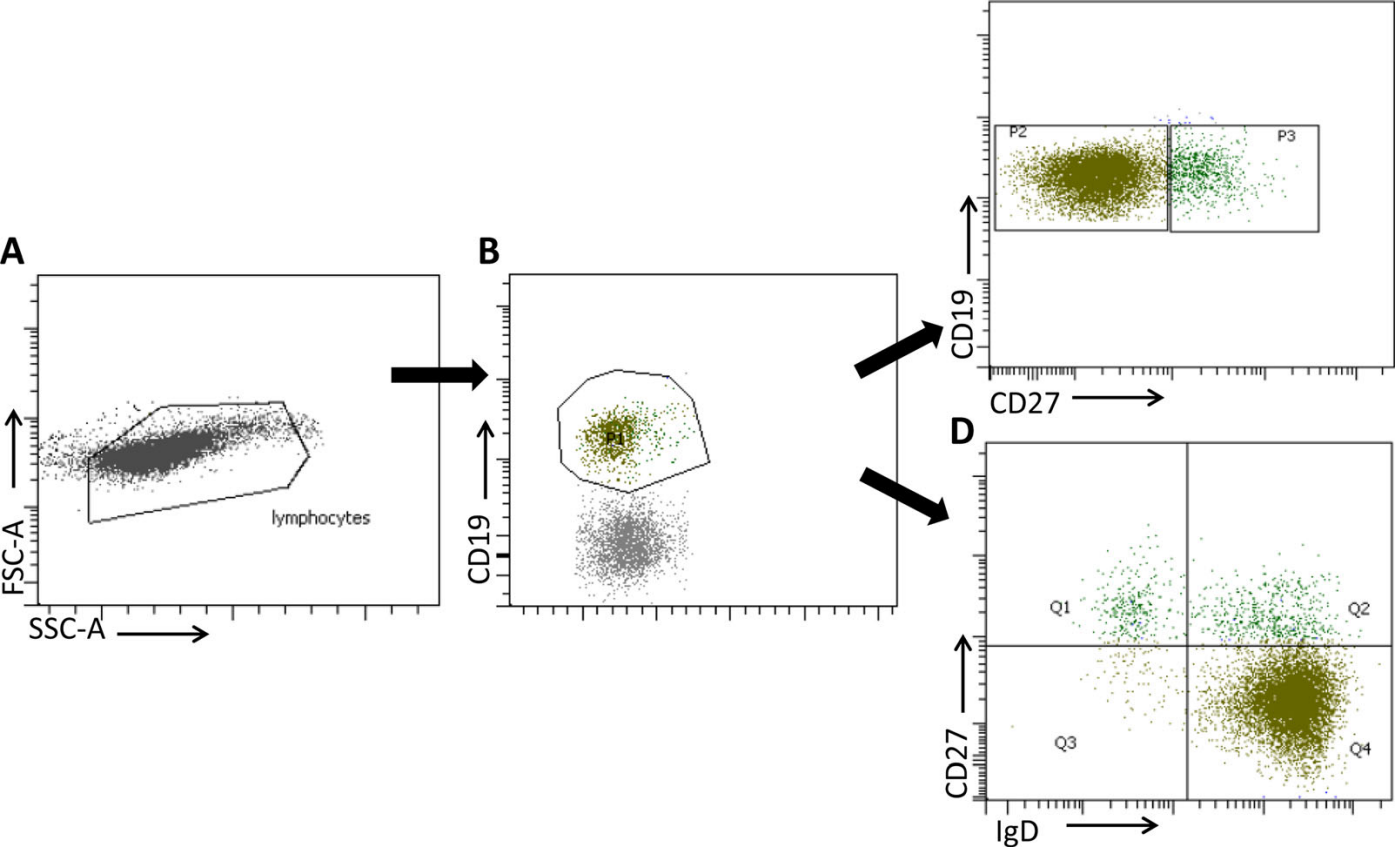
Gating strategy for the analysis of B-cell subsets. Lymphocytes were gated according to forward and side scatter (A). B-cells were identified as CD19-expressing cells in the lymphocyte population (B). A CD27/CD19 dot plot defined total CD27^+^ CD19^+^ memory B-cells (C). The double staining of B-lymphocytes for CD27 and IgD made it possible to determine the percentages of naive B-cells (IgD^+^ CD27^−^), switched memory B-cells (IgD^−^ CD27^+^) and non-switched memory B-cells (IgD^+^ CD27^+^) (D).

Before subject inclusion, a standardized protocol was developed, to prevent inter-center bias. Soluble Ig was eliminated by washing 100 µL aliquots of whole blood three times with cell wash buffer (Becton Dickinson (BD), Rungis, France). The cells were then stained by incubation with monoclonal antibodies directed against CD19 (J3-119, Beckman), CD27 (M-T271, BD) and IgD (Dako R5112 or IA6-2, BD) for 30 min at room temperature. The erythrocytes were lysed with FACS Lysis buffer (BD) or Versalyse (Beckman Coulter), according to the manufacturer's instructions. The cells were washed twice in cell wash buffer (BD) and fixed in a cell fixation solution (BD). B-cell compartment analysis was performed within 24 h of fixation. Absolute numbers were calculated by multiplying the percentage of the subset concerned by the total number of lymphocytes obtained by flow cytometry. All analyses were performed on the cytometer available at the hospital concerned (FACS Canto II Becton Dickinson, Navios or FC500 Beckman Coulter). The gating strategies are explained in Figure 1. The total lymphocyte population was identified on the basis of forward (FSC) and side (SSC) scatter characteristics. B-cells were defined as CD19-expressing cells from the lymphocyte population. We analyzed the expression of IgD and CD27 on CD19^+^ B-cells. Naive B-cells were defined as CD27^−^IgD^+^ cells, non-switched memory B-cells were defined as CD27^+^IgD^+^ cells and switched memory B-cells were defined as CD27^+^IgD^−^ cells.

### Statistical analysis

The data were analyzed with Microsoft Excel® and GraphPad Prism® software. All results are presented as median values and 5th and 95th percentiles. Reference values were established for seven age groups. Before determining the reference intervals, the data were examined to identify outliers. The outlier exclusion was performed according to the Dixon outlier method recommended by the CLSI (Clinical Laboratory and Standards Institute) for non-Gaussian populations [30]. In Dixon's test, if the calculated absolute difference between the suspected outlier and the next closest observation is more than one-third of the absolute difference between the outlier and the furthest observation from the outlier, then the outlier can be rejected. If two or more outliers are suspected, Dixon's rule is applied to the least extreme outlier and the entire group of outliers may be excluded. Following the exclusion of outliers, we calculated the median value and the 5th and 95th percentiles for each age group. When comparison between two groups was necessary, the Mann–Whitney test was used. A *P* value <0.05 was considered significant.

## Results

**Figure 2:**
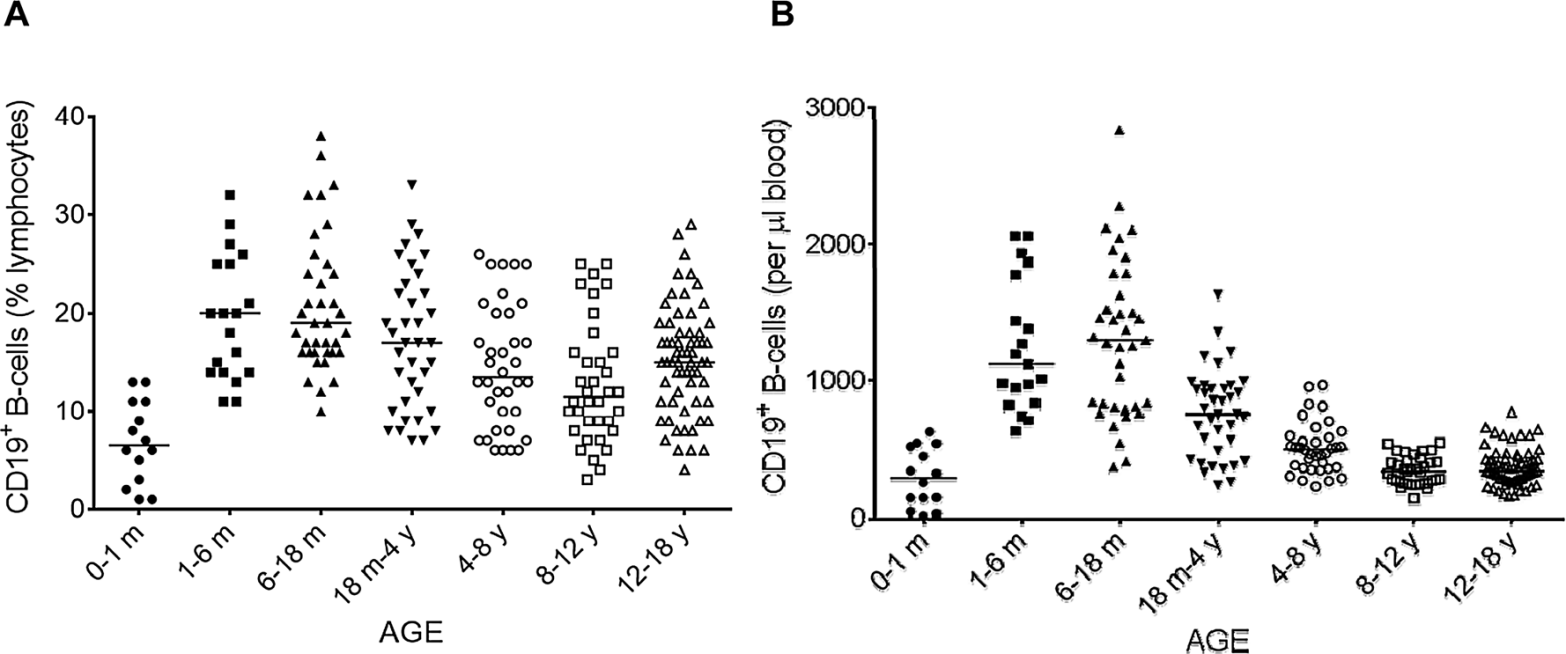
Changes in the percentage (A) and absolute number (B) of total B-cells with age. The proportions of lymphocytes accounted for by total B-cells (CD19^+^) (A) were analyzed by flow cytometry on whole-blood samples. The corresponding absolute numbers (B) were calculated from the absolute numbers of lymphocytes. Solid horizontal lines indicate the median values for each age group. m = month; y = year.

**Figure 3:**
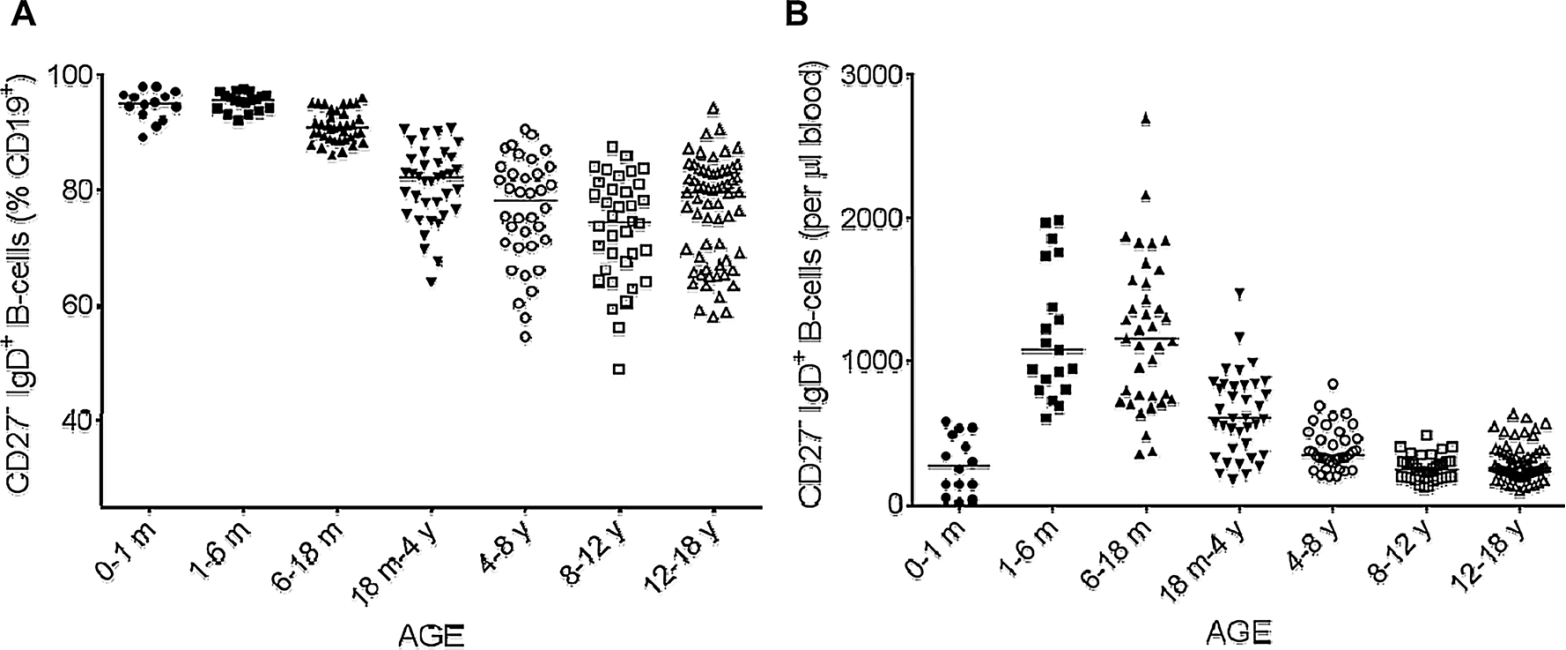
Changes in the percentage (A) and absolute number (B) of naive B-cells according to age. The proportions of naive B-cells (CD27^−^ IgD^+^) (A) among CD19^+^ lymphocytes were analyzed by flow cytometry on whole-blood samples. The corresponding absolute numbers (B) were calculated from the absolute number of B-cells. Solid horizontal lines indicate the median values for each age group. m = month; y = year.

We initially included 292 children, but 50 of these children were excluded according to the Dixon's rule for non-Gaussian populations. B-cell subpopulations were therefore studied in a final population of 242. These children were classified into seven groups on the basis of age: from birth to 1 month of age (*n* = 14), from 1 to 6 months (*n* = 19), from 6 to 18 months (*n* = 37), from 18 months to 4 years (*n* = 37), from 4 to 8 years (*n* = 36), from 8 to 12 years (*n* = 36) and from 12 to 18 years (*n* = 63; Table1). During the first few days of life, B-cell percentage and counts were very low (Fig. 2). Given the relative lymphocytosis observed in infants, the absolute number of B-cells was high between the ages of 1 month and 18 months. It subsequently decreased (Fig. 2B), whereas the percentage of B-cells remained more or less stable after the age of 18 months (Fig. 2A). Naive B-cells continue to express IgD at the cell surface, but they do not yet display CD27 expression, which is induced by antigen-receptor activation in B-cells. There was a significant, large increase in the size of the naive B-cell pool during the first 6 months of life (*P* < 0.0001 for comparison of the 0–1 and 1–6 months age groups), due to the physiological lymphocytosis in infants, but the percentage of naive B-cells among total B-cells was similar for the 0–1 and 1–6 months age groups (*P* = 0.61) (Tables2 and 3, Fig. 3). The percentage of naive B-cells among total B-cells decreased steadily from birth to the age of 12 years. The 1–6 months and 6–18 months age groups displayed the largest decrease in naive B-cell percentage (*P* < 0.0001), and further decreases were observed in the 6–18 months and 18 months to 4 years age groups (*P* < 0.0001). Further slight but significant decreases were observed in the 18 months to 4 years and 4–8 years age groups (*P* = 0.04), followed by a stabilization of naive B-cell percentage at about 70–80%. The apparent slight increase observed in the 12–18 years age group was not significant (*P* = 0.1; Fig. 2A). The absolute number of naive B-cells increased during the first month of life to reach a steady state that was maintained until the age of 18 months, with no significant difference between the values obtained between the ages of 1 month and 18 months (*P* = 0.9). The subsequent rapid decrease in the number of naive B-cells reflects a physiological decrease in the size of the lymphocyte and B-cell populations on one hand, and a decrease in the percentage of naive B-cells on the other. Finally, despite the considerable dispersion of naive B-cell percentage values, particularly for children under the age of 4 years, absolute naive B-cell counts eventually stabilized at about 250 cells per microliter of blood, with lower levels of inter-individual variability (Fig. 3B).

**Table 1:** Demographic information for the 242 healthy children tested

Age group	Median age	Number of children
0–1 month	3.0 days	14
1–6 months	1.9 months	19
6–18 months	11.5 months	37
18 months–4 years	3.1 years	37
4–8 years	6.1 years	36
8–12 years	10.3 years	36
12–18 years	14.7 years	63

**Figure 4:**
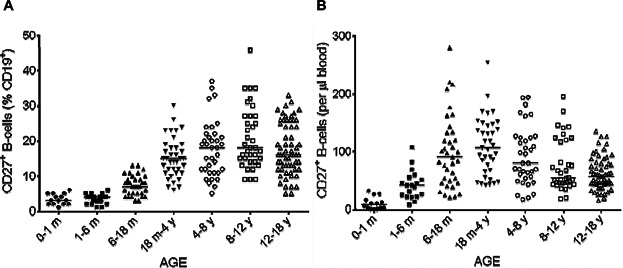
Changes in the percentage (A) and absolute number (B) of total memory B-cells with age. The proportions of total memory B-cells (CD27^+^) (A) among CD19^+^ lymphocytes were analyzed by flow cytometry on whole-blood samples. The corresponding absolute numbers (B) were calculated from the absolute number of B-cells. Solid horizontal lines indicate the median values for each age group. m = month; y = year.

**Figure 5:**
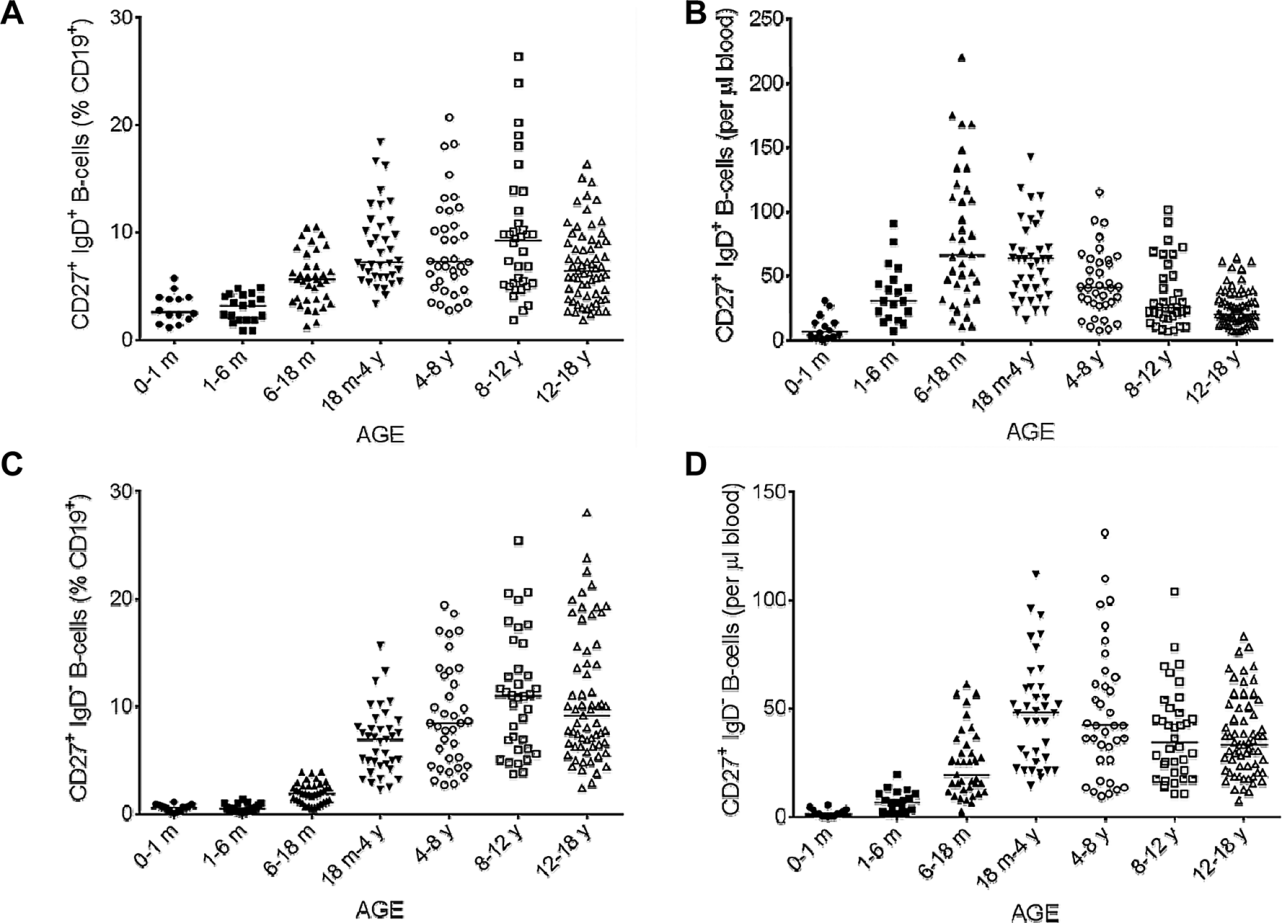
Changes in the percentage and absolute number of memory B-cells subsets with age. The proportions of non-switched memory B-cells (CD27^+^ IgD^+^) (A) and switched memory B-cells (CD27^+^ IgD^−^) (C) among CD19^+^ lymphocytes were analyzed by flow cytometry on whole-blood samples. The corresponding absolute numbers (B and D) were calculated from the absolute number of B-cells. Solid horizontal lines indicate the median values for each age group. m = month; y = year.

We analyzed the age-dependent distribution of the various mature peripheral B-cell subsets by double-labeling for CD27 and IgD. Surface CD27 expression defines memory B-cells, a population comprising two different subsets: non-switched memory B-cells or marginal zone B-cells, which continue to express IgD on their surface (CD19^+^ CD27^+^ IgD^+^) and switched memory B-cells, which are characterized by a loss of IgD expression (CD19^+^ CD27^+^ IgD^−^). The proportion of total memory B-cells (corresponding to both subsets; Fig. 4A) gradually increased, reaching a peak between the ages of 4 and 8 years (median: 18.4%, Table2), whereas the absolute count of CD27^+^ B-cells peaked between the ages of 6 months and 4 years, subsequently decreasing and then stabilizing (Fig. 4B, Table2). The percentage of non-switched and switched memory B-cells among total B-cells increased during infancy, but with different time courses (Table2, Fig. 5A and C). Switched memory B-cells were almost undetectable between birth and the age of 6 months, whereas non-switched memory B-cells were already present, albeit at very low percentages, in the first 6 months of life. We also observed a gradual slight increase in the percentage of CD27^+^ IgD^+^ B-cells, whereas the percentage of CD27^+^ IgD^−^ B-cells increased more rapidly between the 6–18 months and 18 months to 4 years age groups, reaching a peak at the age of 8–12 years before stabilizing. We also observed differences in absolute numbers between these two subsets of memory B-cells (Table3, Fig. 5B and D). During the increase in total B-cell numbers between 1–6 months and 18 months to 4 years, the absolute numbers of non-switched memory B-cells increased similarly and then decreased, but this decrease occurred later than that in the number of total B-cells. The decrease was highly significant for total B-cells between 6–18 months and 18 months to 4 years (*P* < 0.0001) whereas the decrease in the numbers of CD27^+^ IgD^+^ B-cells was not significant between 6–18 months and 18 months to 4 years (*P* = 0.32) but was significant between 18 months to 4 years and 4–8 years (*P* = 0.009). Conversely, the absolute number of switched memory B-cells gradually increased, peaking between the ages of 18 months and 4 years, but remaining stable thereafter, with no significant decrease. The large differences within age groups may be accounted for by variations in genetic background, antigen exposure, or environmental factors influencing B-cell maturation. However, it is important to have access to “normal” ranges of these values, to make it possible to identify children with an impaired B-cell phenotype.

**Table 2:** Percentages of B-cell subsets by age group (%)

Age no. of individuals	0–1 m (*n* = 14)	1–6 m (*n* = 19)	6–18 m (*n* = 37)	18 m–4 y (*n* = 37)	4–8 y (*n* = 36)	8–12 y (*n* = 36)	12–18 y (*n* = 63)
Lymphocytes	34.0 (18.8–52.0)	67.0 (46.0–74.6)	59.0 (38.2–71.2)	47.8 (28.4–65.9)	39.0 (27.9–54.4)	37.0 (17.3–48.8)	31.6 (16.5–50.4)
CD19^+^	6.5 (0.9–13.0)	19.7 (11.1–29.3)	19.0 (12.4–33.6)	16.8 (7.6–28.2)	13.5 (6.1–25.2)	11.3 (4.8–24.3)	15.2 (6.5–24.0)
CD19^+^ CD27^+^	3.0 (1.5–5.4)	3.5 (1.2–5.1)	7.3 (3.5–12.2)	14.7 (7.0–24.3)	18.4 (8.1–33.3)	17.8 (9.0–35.0)	16.0 (7.0–29.0)
CD27^−^IgD^+^	95.0 (90.5–98.0)	95.5 (93.1–97.3)	91.0 (87.3–95.1)	82.3 (69.2–90.5)	78.2 (59.7–88.4)	74.4 (58.5–84.6)	79.6 (61.6–87.4)
CD27^-^IgD^−^	1.5 (0.4–4.2)	1.0 (0.3–3.0)	1.0 (0.4–2.7)	3.1 (1.2–8.3)	5.1 (1.7–13.2)	5.0 (2.3–11.9)	4.9 (1.4–13.0)
CD27^+^IgD^+^	2.6 (1.2–5.1)	3.1 (0.8–4.7)	5.6 (2.4–9.9)	7.2 (4.6–16.3)	7.3 (3.1–18.0)	9.2 (3.0–21.1)	6.4 (2.6–13.4)
CD27^+^IgD^−^	0.5 (0.1–0.9)	0.4 (0.1–1.0)	1.8 (0.6–3.7)	6.9 (2.7–12.5)	8.4 (2.9–17.4)	11.0 (4.4–20.5)	9.1 (4.0–21.2)

The percentages of lymphocytes (as a percentage of total leukocytes), total CD19^+^ B-cells (as a percentage of total lymphocytes) and B-cell subsets (as a percentage of total CD19^+^ B-cells) are shown for each age group, as medians (upper line) and as the corresponding 5th and 95th percentiles (lower line). m = months; y = years.

**Table 3:** Absolute numbers of B-cell subsets by age group (/µL blood)

Age no. of individuals	0–1 m (*n* = 14)	1–6 m (*n* = 19)	6–18 m (*n* = 37)	18 m–4 y (*n* = 37)	4–8 y (*n* = 36)	8–12 y (*n* = 36)	12–18 y (*n* = 63)
Lymphocytes	3,922 (2,195–6,218)	5,800 (3,944–8,030)	4,791 (3,297–7,820)	3,900 (2,373–7,249)	2,810 (1,761–3,873)	2,209 (1,445–3,725)	2,057 (1,379–3,663)
CD19^+^	292 (24–580)	1,121 (712–2,059)	1,300 (523–2,149)	759 (319–1,244)	504 (273–860)	336 (219–509)	340 (193–628)
CD19^+^CD27^+^	8 (1–29)	42 (13–85)	91 (25–217)	107 (45–175)	80 (23–185)	54 (31–152)	57 (26–115)
CD27^−^ IgD^+^	271 (23–553)	1,080 (677–1,968)	1,161 (461–1,930)	607 (212–1,027)	342 (203–648)	241 (128–403)	254 (126–546)
CD27^−^ IgD^−^	2 (0–17)	11 (3–31)	11 (4–28)	20 (10–56)	23 (8–74)	18 (7–35)	16 (6–42)
CD27^+^IgD^+^	6 (1–27)	30 (13–78)	66 (14–170)	63 (23–113)	40 (7–91)	25 (8–81)	20 (7–56)
CD27^+^ IgD^−^	1 (0–4)	6 (1–13)	19 (7–57)	48 (20–93)	42 (11–103)	34 (13–72)	33 (12–69)

The absolute numbers of lymphocytes, total CD19^+^ B-cells and of each B-cell subset per µL blood are shown for each age group, as medians (upper line) and as the corresponding 5th and 95th percentiles (lower line). m = months; y = years.

## Discussion

We established normal values and percentages for total, non-switched and switched memory B-cells in a large pediatric cohort of healthy children, through a national, multicenter study. Most of the changes in B-cell subpopulations occur during childhood, particularly during the first 5 years of life, when children encounter a multitude of different antigens. At least six single-center studies on this theme have been published [12–14,26–28]. These studies proposed reference intervals for B-cell subsets and described the age-dependent development of memory B-cells. We carried out a national study and validated common reference intervals, to prevent confusion due to the use of different laboratory-specific reference values. Common reference intervals are widely used for children, because ethical issues limit access to samples from healthy children [31–33]. The use of a multicenter approach, with the cohort recruited from a number of different laboratories, made it possible to include a large number of healthy children and, therefore, to achieve an acceptably high level of statistical confidence. Differences between ethnic groups have been reported. The establishment of national reference values for a large cohort representative of the entire population is therefore required for the interpretation of blood analyses. The adoption of this unique set of reference tables by laboratories should help physicians to interpret results and to diagnose immunological conditions. The most recent PID and CVID classifications are multicentric [17,24], and immunological investigations should now be standardized and shared between hospitals, for the constitution of homogeneous patient cohorts.

We explored memory B-cells identified on the basis of their CD27 expression and the differential expression of IgD, which is often used to separate switched and non-switched memory B-cells. Some studies have also identified B-cell subpopulations on the basis of their IgM expression, defining a subpopulation of CD27^+^ IgM^+^ IgD^−^ B-cells and a subpopulation of CD27^+^ IgM^+^ IgD^+^ marginal zone B-cells [13,26]. However, the consideration of IgM status results in considerable variability and a lack of reproducibility between the different laboratories. Indeed, the intensity of IgM expression is extremely variable, potentially accounting for this phenomenon. For the establishment of a robust standard protocol and to reduce analytical variability, we decided not to consider IgM expression in our method. IgM-only B-cells (CD27^+^ IgM^+^ IgD^−^) and IgD-only B-cells (CD27^+^ IgM^−^ IgD^+^) constitute only minor populations in the B-cell compartment [10]. This approach therefore introduced very little heterogeneity into the observed subsets. Modifications to the memory B-cell compartment defined on the basis of CD27 and IgD expression only have already been associated with mutations in genes affecting late B-cell development (e.g., CD27 and CD21) [34–37]. We observed a gradual decrease in the percentage and absolute numbers of naive B-cells, beginning at the age of 18 months. These results are similar to those obtained in four other studies [12–14,26]. Interestingly, whereas the percentage of naive B-cells was similar in the 0–1 and 1–6 months groups, naive B-cells absolute numbers differed significantly between these two groups, due to the physiological lymphocytosis occurring in infants from the age of 1 month. This observation highlights the need for accurate age-matching for reference frequencies and absolute numbers, particularly during early infancy. The number of naive B-cells reflects the capacity for normal homeostatic proliferation of B lymphocytes and for normal antigen-induced differentiation in germinal centers or the marginal zone.

Switched memory B-cells, expressing CD27, have undergone somatic hypermutation and class-switch recombination in germinal centers, where the selection of memory B-cells is driven by encounters with T-dependent antigens [9]. CD27 is a member of the TNFR family [38] and it is widely associated with B-cell activation and differentiation [10,11]. A shift from naive B-cells to memory B-cells was observed during infancy. As previously described, the absolute number of switched memory B-cells peaked between the ages of 18 months and 4 years, before stabilizing [12,13]. All the available reference values for the percentage of CD27^+^ IgD^−^ B-cells have reported a steady increase until the age of 18 years and into adulthood [12–14,26,27]. The role of CD27 remains a matter of debate in the memory B-cell field [7,39], but decreases in CD27 expression and isotype-switched cells may be indicative of an impaired germinal center reaction, as highlighted in some PIDs [34,40]. The population of CD19^+^ CD27^+^ IgD^+^ cells, defined in our study as non-switched memory B-cells, corresponds to circulating marginal zone B-cells [41,42]. These cells are involved in T-independent responses and play a crucial role in the control of infections due to encapsulated bacteria [2,43]. The reported pattern of development of this small population with age differs slightly between studies. Some investigators have reported fluctuations in the absolute number of CD27^+^ IgD^+^ cells throughout childhood [12,13]. By contrast, we and others have reported a gradual increase in the percentage and absolute number of non-switched memory B-cells between early childhood and adolescence [14,28]. This increase may reflect the widely accepted immaturity of the T-independent antibody response in the first few years of life [44,45]. Moreover, CD27^+^ IgD^+^ memory B-cells slowly accumulate mutations, diversifying the B-cell repertoire, during this period of life [45,46].

Memory B-cell heterogeneity is still being investigated. Indeed, a distinct memory population, double-negative for IgD and CD27, has been described as presenting a pattern of somatic hypermutation consistent with antigen-driven selection [47–49]. The percentage of this minor population increases during the first 6 years of life, remaining stable thereafter. Studies of adult cohorts have confirmed the stability of the CD27^+^ IgD^−^ population [12]. This population has been quantified and its phenotype described, but its origin and physiological role remain to be elucidated. The low percentage of somatic mutation in switched CD27^+^ B-cells suggests that these cells may emerge as a first wave of memory B-cells [50,51]. This population seems to be expanded in adult systemic autoimmune diseases and chronic infectious diseases [48,52–55]. No study has yet been published concerning this population in children. Pediatric reference intervals may facilitate the detection of abnormal development of this B-cell population in the first few years of life and may reveal associations with pathological contexts. In conclusion, we determined national reference values for various B-cell subsets, which have been adopted by all the participating laboratories. These reference intervals facilitate the interpretation of immunological investigations and characterization of the B-cell defects seen in PIDs. Nevertheless, serum Ig levels are often insufficient to guide the diagnosis and genetic analysis of these defects [56]. Our global approach, based on the memory and naive B-cell subsets, can be used to separate patients into subgroups and to propose an appropriate diagnosis strategy for each subgroup. This may lead to the suspicion of several deficiencies, most affecting late stages of B-cell development (e.g., CD27 or CD21 deficiencies), or ICF syndrome [16,20,36]. The common reference values provided here can be used to constitute homogeneous national cohorts, thereby facilitating the detection of immunologic and phenotypic associations, particularly for PADs.
